# Tethered Cord Syndrome

**DOI:** 10.5811/cpcem.2019.4.42536

**Published:** 2019-07-03

**Authors:** Shawn Catmull, John Ashurst

**Affiliations:** Kingman Regional Medical Center, Department of Emergency Medicine, Kingman, Arizona

## Abstract

Tethered spinal cord syndrome refers to signs and symptoms of motor and sensory dysfunction related to increased tension on the spinal cord due to its abnormal attachment; it has classically been associated with a low-lying conus medullaris. Treatment is primarily surgical and has varying degrees of results. Although rarely diagnosed in the emergency department, the emergency physician must be aware of the disease in patients presenting with signs and symptoms concerning for cauda equina syndrome.

## CASE PRESENTATION

**Image 1:**
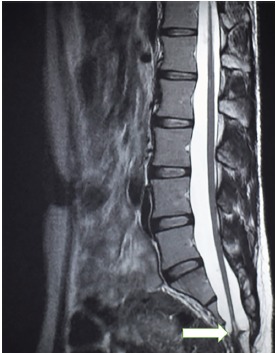
Sagittal view of a T2-weighted magnetic resonance image of the lumbar spine showing the conus medullaris of the tethered cord reaching to inferior edge of the second sacral vertebrae (arrow).

**Image 2:**
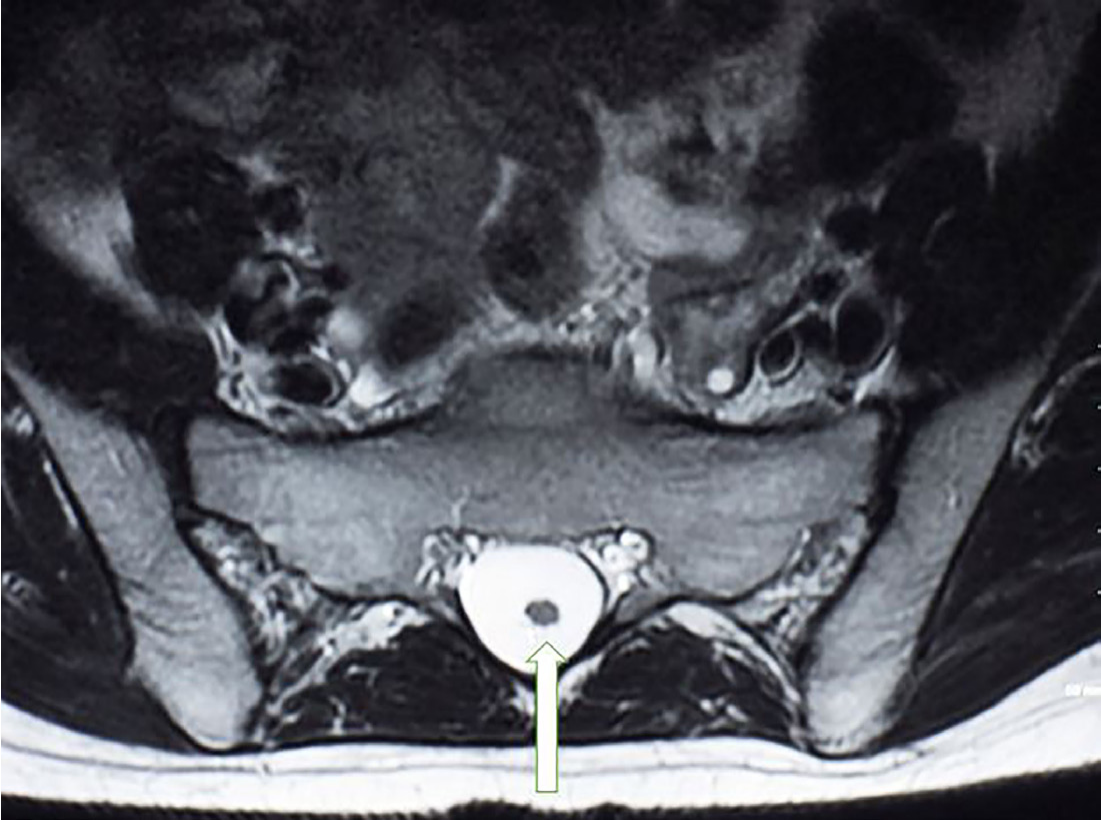
Axial view of the T2-weighted magnetic resonance image showing the continuation of the spinal cord into the sacral region (arrow).

A 30-year-old male presented to the emergency department with a three-day history of low back pain associated with urinary incontinence. His past medical history was significant for numerous urinary tract infections and bilateral hydronephrosis with associated mega-ureters status post ureteral stenting. Physical examination revealed tenderness to palpation along the paravertebral musculature of the lower back, normal muscle strength and tone, normal sphincter tone and no paresthesia along any dermatome. Magnetic resonance imaging (MRI) of the lumbar spine was obtained and depicted a dorsally positioned spinal cord segment within the spinal canal that continued to the position of the fifth lumbar and first sacral vertebrae (Images 1 and 2). The patient’s case was discussed with the neurosurgical services and he underwent surgical decompression as an outpatient. The patient had complete resolution of his urinary symptoms following the procedure.

## DIAGNOSIS

Tethered spinal cord syndrome (TCS), first described in 1857, is a neurological disorder caused by an abnormal attachment of the spinal cord to surrounding tissues.^1^,^2^ TCS can be caused by congenital (primary) or acquired (secondary) disorders.^1^,^2^ Congenital disorders can occur anytime during embryologic development.^1^,^2^ Acquired TCS can occur from lipomas, abnormal dural tracts, infections, or trauma.^1^,^2^ The most common physical exam finding in adult patients with TCS is low back pain with flexion, but other neurologic findings including weakness, paresthesia, gait abnormalities, and incontinence may occur.^1^–^3^ In those suspected of TCS, the spine should be examined for any signs of spina bifida and scoliotic deformities and the feet should be examined for any signs of clubbing.^1^,^2^ MRI is currently the gold standard for diagnosis, and neurosurgical intervention is the treatment of choice.^1^,^2^

**CPC-EM Capsule**

What do we already know about this clinical entity?

Tethered cord syndrome is a rare cause of back pain that is caused by an abnormal attachment of the spinal cord to surrounding tissues.

What is the major impact of the image(s)?

The image depicts the conus medullaris below the typical anatomical location that is expected by the emergency physician (EP). The EP should be aware of anatomical variants for the future treatment of patients.

How might this improve emergency medicine practice?

The EP should keep a broad differential when faced with a patient with back pain and frequent urinary tract infections.
